# Unlocking Osteoporosis Diagnosis: Unveiling the Potential of MRI-Based Vertebral Bone Quality Score

**DOI:** 10.7759/cureus.82154

**Published:** 2025-04-12

**Authors:** Divya Pabbisetti, Anantaram Gudipati, Amber Papalkar, Sandeep Ponnaganti, Harshitha Shanbhag, Akanksha Bhashyakarla, Kiran Kumar Sailagundla, Pujitha Innamuri

**Affiliations:** 1 Department of Radiology, Krishna Institute of Medical Sciences, Hyderabad, IND; 2 Department of Radiology, Bala Gangadharanatha Swamiji (BGS) Medical College and Hospital, Bangalore, IND; 3 Department of Radiology, Sacred Heart Hospital, Pensacola, USA

**Keywords:** bone mineral density (bmd), dual-energy x-ray absorptiometry (dexa), osteopenia, osteoporosis, vertebral bone quality (vbq) score

## Abstract

Introduction: Osteoporosis is the most common skeletal disease in humans. The current gold standard technique to diagnose osteoporosis is the measurement of bone mineral density (BMD) using dual-energy x-ray absorptiometry (DEXA). However, DEXA overestimates BMD when there are degenerative changes. Vertebral bone quality (VBQ) score may serve as a supplementary technique.

Aim: To explore the value of VBQ score in diagnosing osteoporosis in patients with and without degenerative changes. To formulate a cut-off VBQ score for diagnosing osteoporosis.

Materials and methods: A retrospective study was conducted using the data of 112 patients who underwent radiographs, MR imaging, and DEXA scans of the lumbar spine in our hospital over a period of one year from July 2023 to 2024. The patients were divided into degenerative and control groups based on radiographic findings. VBQ score was calculated as the ratio of mean signal intensity (SI) of L1 to L4 vertebral bodies and signal intensity (SI) of cerebrospinal fluid (CSF) at L3 on T1. Demographic data, BMD, and T-score were recorded. Pearson correlation coefficient was used to compare the VBQ score with the BMD and T-score. The VBQ score threshold was obtained and compared with the efficacy of osteoporosis diagnosis based on DEXA.

Results: The degenerative group was older than the control group (70.1 vs. 59.8, P = 0.001). The VBQ score of the control group suggested a higher correlation with BMD value and T-score (r = −0.506 and −0.520, respectively). The BMD value and T-score in the degenerative group were higher (P < 0.05). Receiver operating characteristic (ROC) curve analysis showed that the VBQ score had good predictability for osteoporosis (area under the curve (AUC) = 0.804), with 71.4% sensitivity and 81.8% specificity in the control group. Based on the T-score, there was no significant difference in the prevalence of osteoporosis between the degenerative and control groups (18.5% vs. 24.1%, P= 0.46); based on the VBQ score, the prevalence in the degenerative group was significantly higher (55.5% vs. 40.9%, P= 0.025).

Conclusion: MRI-based VBQ score is a simple, easy-to-calculate tool and does not require exposure to ionizing radiation to analyze bone quality. VBQ scores potentially overcome the shortcomings of DEXA for diagnosing osteoporosis, especially in older patients with more degenerative changes, where setting DEXA may give spurious results. VBQ score may be used as a supplementary tool along with DEXA. However, MRI is an expensive investigation, and its utility as a diagnostic tool for osteoporosis in the absence of other indications, such as degenerative disc disease, may not be justified. Further studies with larger sample sizes are required to formulate a VBQ score threshold cut-off value for osteoporosis diagnosis.

## Introduction

Osteoporosis is the most common skeletal disease of humans, characterized by low bone mass and compromised bone strength [1]. The current gold standard tool for the diagnosis of osteoporosis is dual-energy x-ray absorptiometry (DEXA) [2]. WHO defines osteoporosis as a DEXA-based T score <−2.5 [3].

DEXA overestimates bone density in the presence of degeneration, such as scoliosis, osteophytosis, reduction in disc height, end-plate sclerosis, aortic atherosclerotic calcification, etc. [4]. MRI-based vertebral bone quality (VBQ) score is a relatively new method for assessing bone quality.

## Materials and methods

Aims and objectives

To explore the value of VBQ score in diagnosing osteoporosis in patients with and without degenerative changes. To formulate a cut-off VBQ score for diagnosing osteoporosis.

Methodology

This is a retrospective study done over a period of 15 months from July 2023 to October 2024 after obtaining the approval of the Ethics committee of our hospital. The pooled sensitivity of the VBQ score in diagnosing osteoporosis was reported as 81% as per the meta-analysis done by Chen et al [5]. Using a 95% confidence interval and a 10% precision level, the minimum required sample size was calculated to be 118.

Patients with radiographs, MR imaging, and DEXA scans of the lumbar spine done in our institute not more than six months apart were included in our study. Our analysis included 120 patients. The exclusion criteria are patients with prior lumbar spine surgery, a history of spondyloarthropathy, metabolic bone disease, radiotherapy, a spinal tumor or infection, and >2 lumbar vertebral fractures. 

**Figure 1:**
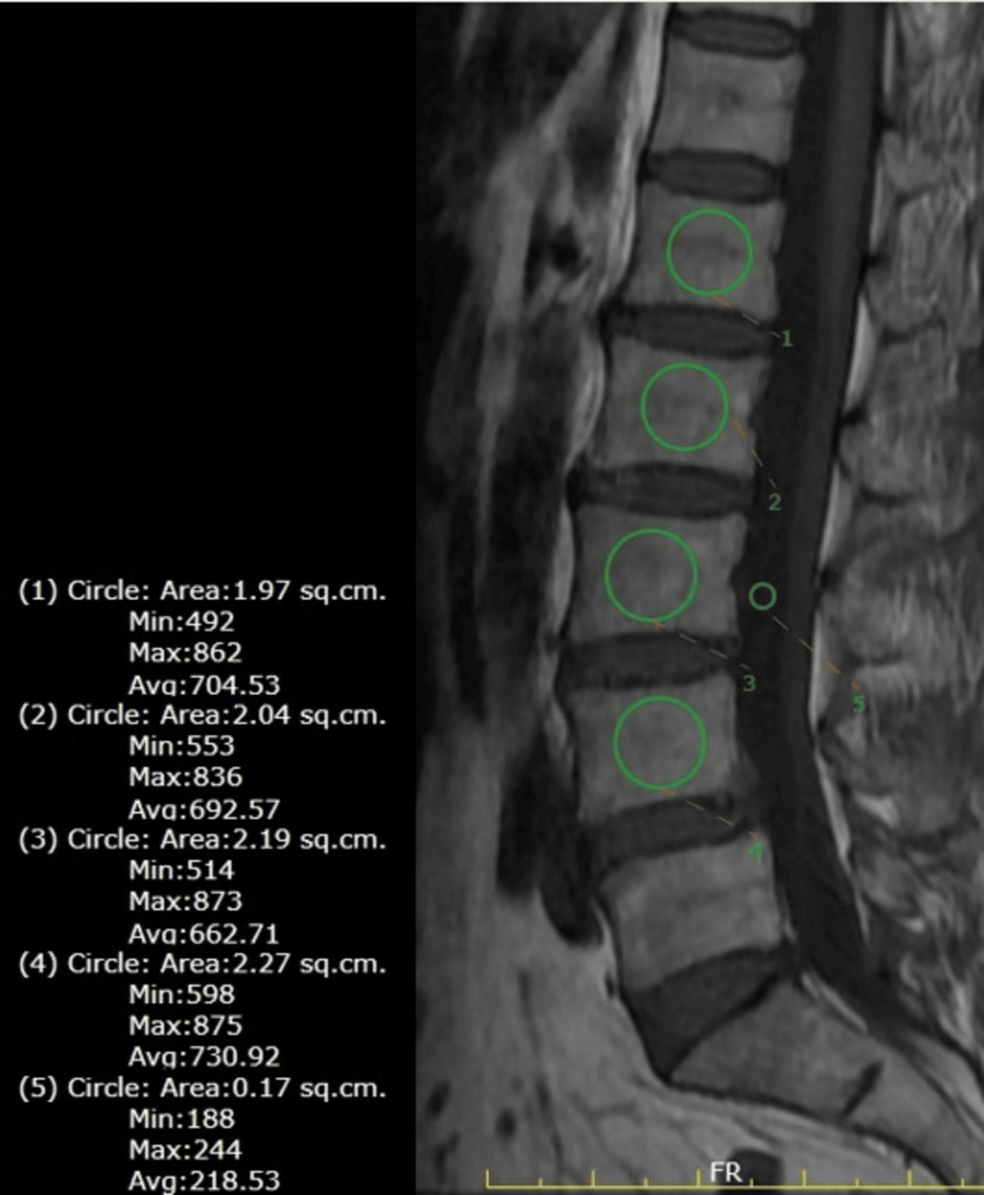
T1-weighted mid-sagittal image showing equal-sized ROIs drawn within the L1-L4 vertebral body and another ROI drawn within the anterior subarachnoid CSF space at the L2 level. ROI: region of interest, CSF: cerebrospinal fluid.

Our patients had MR imaging of the lumbar spine done on either a 1.5T or 3T scanner. On the mid-sagittal section on routine T1-sequences, circular region of interests (ROIs) were drawn entirely within the cancellous bone, avoiding Modic changes, vertebral venous plexus, bone islands, etc., in L1-L4 vertebral bodies and signal intensity (SI) values were recorded. Another ROI was drawn within the anterior subarachnoid space within the spinal canal at the L3 level and its signal intensity value was recorded (Figure 1). VBQ score was calculated as the ratio between the mean SI (L1-L4) and CSF SI at L3. 

If there was scoliosis, parasagittal sections were used. If there was no CSF in the anterior subarachnoid space at the L3 level due to canal stenosis, the level above or below was used. If there was an acute or chronic fracture, that specific vertebral level was excluded. All our patients had a DEXA scan on a Wipro GE scanner-Prodigy series (Wipro GE Medical Systems, Madison, WI). Demographic data, BMI, lumbar spine DEXA-based T-score, and VBQ score were recorded for every patient.

Based on radiographic findings, these patients were dichotomized into degenerative and control groups. Categorization into degenerative groups required the presence of any of these features at least three vertebral levels: Scoliosis (Cobb angle > 10 degrees), intervertebral disc space narrowing, end-plate sclerosis, and osteophytosis [6]. Two experienced radiologists independently did VBQ score analysis and categorization into degenerative and control groups.

Statistical analysis

Statistical analysis was done using SSPS version 23 (IBM Corp., Armonk, New York, USA). Pearson correlation coefficient was used to analyze the correlation between BMI, T-score, the bone mineral density (BMD) value, and the VBQ score. A receiver operating characteristic curve (ROC) was used to analyze the VBQ threshold score for diagnosing osteoporosis and osteopenia.

## Results

The age range of our study population was 31-88 years. The degenerative group was older than the control group (mean age 70.1 vs. 59.8 years, P = 0.001). Most of the patients in our study were women (89.7%). Group statistics are tabulated in Table 1. The VBQ score correlated negatively with BMI in both degenerative and control groups, with a statistically significant P value (0.02 in the degenerative group and 0.01 in the control group).

**Table 1:** Group statistics-degenerative and control groups. BMI: body mass index; VBQ: vertebral bone quality.

	Degenerative group (mean ± SD)	Control group (mean ± SD)	P-value
Age (years)	70.13 ± 9.270	59.81 ± 11.839	<0.001
BMI	29.17 ± 5.97	27.26 ± 5.33	0.099
L1-L4 BMD	1.10004 ± 0.259	0.99926 ± 0.219	0.028
L1-L4 T-score	-0.761 ± 2.11	-1.529 ± 1.75	0.038
VBQ score	3.3310	3.1574	0.015

The BMD value and T-score in the degenerative group were higher (P < 0.05). The VBQ score correlated negatively with T-score and BMD in both degenerative and control groups, but the correlation in the control group was much stronger (Table 2).

**Table 2:** Correlation between VBQ score and DEXA measures in degenerative and control groups. VBQ: vertebral bone quality; DEXA: dual-energy x-ray absorptiometry, BMD: bone mineral density.

	Degenerative group (N = 58) VBQ score	P-value	Control group (N = 62) VBQ score	P-value
BMD	Pearson correlation coefficient: −0.217	0.115	Pearson correlation coefficient: −0.506	0.000
T-score	Pearson correlation coefficient: −0.202	0.143	Pearson correlation coefficient: −​​​​​​​0.502	0.000

**Figure 2:**
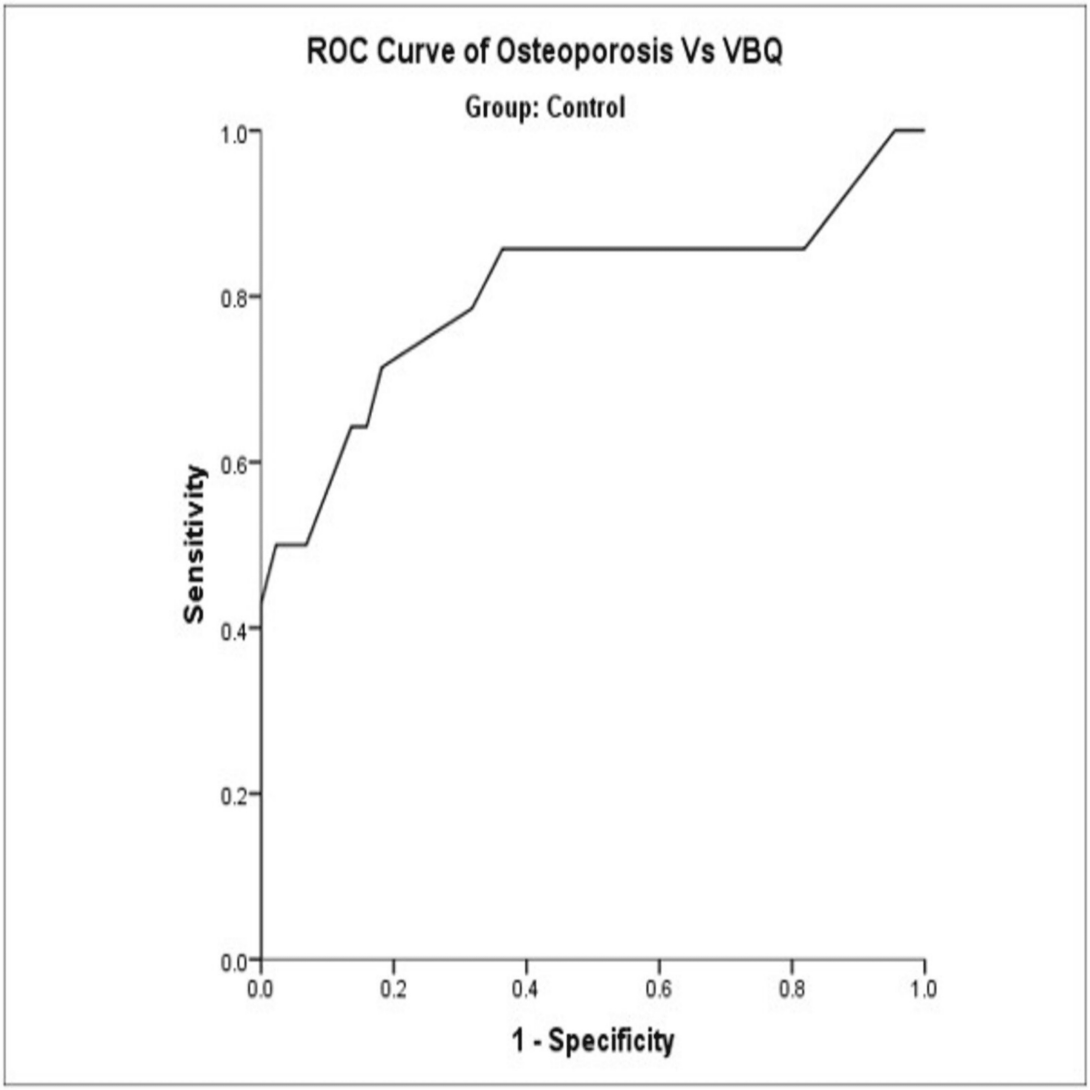
Receiver operating curve (ROC) of osteoporosis vs VBQ (control group). VBQ: vertebral bone quality.

ROC curve analysis showed that the VBQ score had good predictability for osteoporosis (area under the curve (AUC) = 0.804) in the control group. With a VBQ score threshold of 3.55 in the control group, the sensitivity of osteoporosis diagnosis was 71.4% and the specificity was 81.8% (Figure 2, Table 3).

**Table 3:** Sensitivity and specificity of the VBQ score using a threshold of 3.55 based on the ROC curve. The test result variable(s): VBQ has at least one tie between the positive actual state group and the negative actual state group. Statistics may be biased. ^a^Group = control; ^b^under the nonparametric assumption; ^c^null hypothesis: true area = 0.5. VBQ threshold: 3.55; sensitivity: 71.4%; specificity: 81.8%. VBQ: vertebral bone quality; ROC: receiver operating curve.

Area under the curve^a^				
Test result variables			Confidence level	
Area	Std error^b^	Asymptotic sig^c^	Lower bound	Upper bound
0.804	0.083	0.001	0.640	0.967

With a VBQ score threshold of 3.25, the sensitivity of osteoporosis diagnosis was 85.7% and the specificity was 59.1% (Table 4).

**Table 4:** Based on the coordinates of the ROC curve, between the VBQ cut-off scores of 3.05 and 3.55, the sensitivity and specificity of osteoporosis diagnosis range between 85.7-71.4% and 47.7-81.8%, respectively. The test result variable(s): VBQ has at least one tie between the positive actual state group and the negative actual state group. ^a^Group = control. ^b^The smallest cut-off value is the minimum observed test value minus 1, and the largest cut-off value is the maximum observed test value plus 1. All the other cut-off values are the averages of two consecutive ordered observed test values. ROC: receiver operating curve; VBQ: vertebral bone quality.

Coordinates of the curve^a^				
Test result variable(s)				
Positive if greater than or equal to^b^	Sensitivity	1 - Specificity	Specificity	Sum (sensitivity + specificity)
0.700	1.000	1.000	0.000	1.000
2.050	1.000	0.977	0.023	1.023
2.550	1.000	0.955	0.045	1.045
2.750	0.929	0.886	0.114	1.042
2.850	0.857	0.818	0.182	1.039
2.950	0.857	0.614	0.386	1.244
3.050	0.857	0.523	0.477	1.334
3.150	0.857	0.455	0.545	1.403
3.250	0.857	0.409	0.591	1.448
3.350	0.857	0.364	0.636	1.494
3.450	0.786	0.318	0.682	1.468
3.550	0.714	0.182	0.818	1.532
3.650	0.643	0.159	0.841	1.484
3.800	0.643	0.136	0.864	1.506
3.950	0.500	0.068	0.932	1.432
4.050	0.500	0.023	0.977	1.477
4.200	0.429	0.000	1.000	1.429
4.400	0.286	0.000	1.000	1.286
4.550	0.214	0.000	1.000	1.214
4.650	0.143	0.000	1.000	1.143
4.800	0.071	0.000	1.000	1.071
5.900	0.000	0.000	1.000	1.000

**Figure 3:**
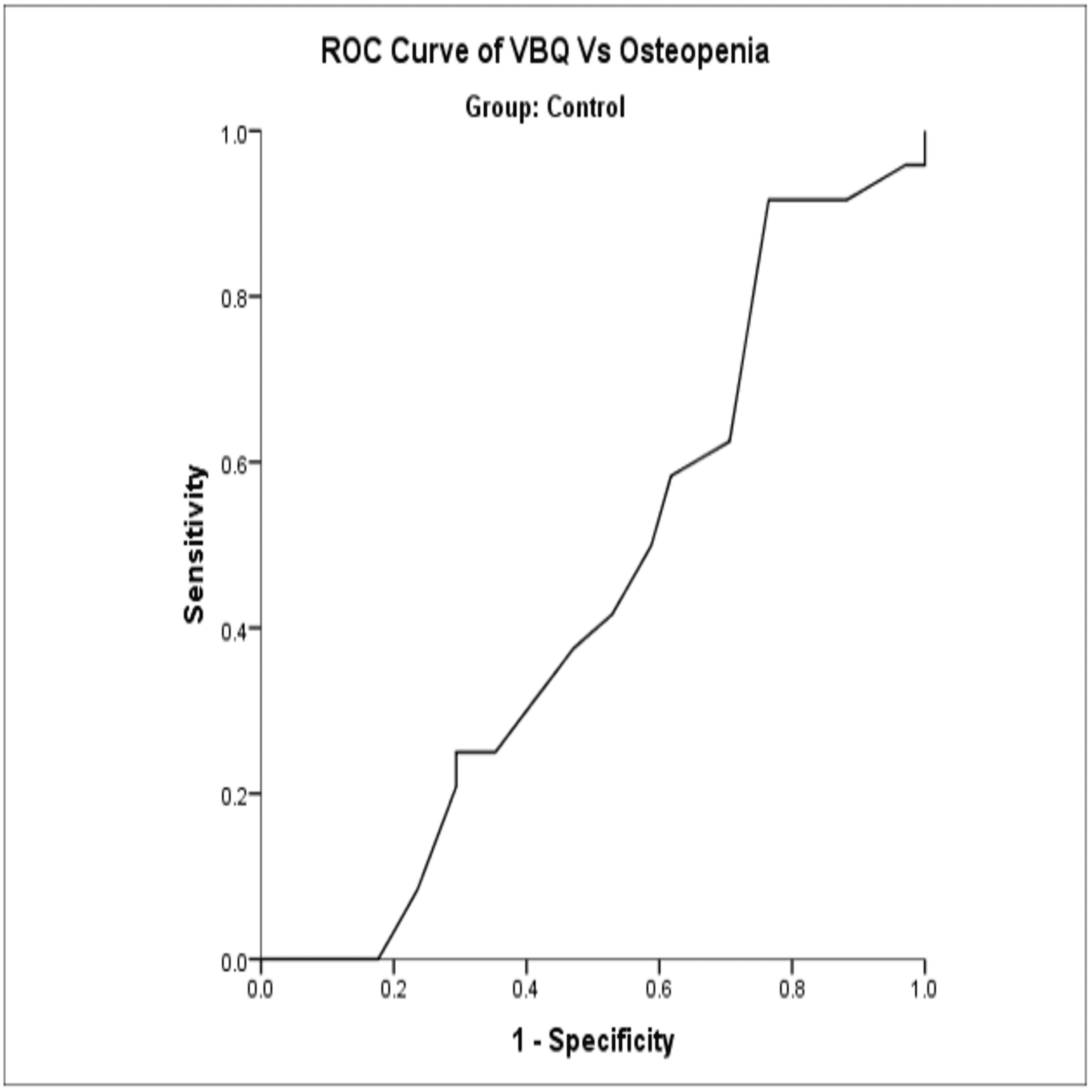
ROC curve of osteopenia vs VBQ score in the control group. ROC: receiver operating curve; VBQ: vertebral bone quality.

VBQ score as a diagnostic tool for osteopenia using a cut-off score of 2.85 showed good sensitivity but poor specificity (Figure 3, Table 5).

**Table 5:** Based on the ROC curve analysis, using a VBQ score threshold of 2.85 for diagnosing osteopenia, there is a good sensitivity of 91.7% but a poor specificity of 23.5%. The test result variable(s): VBQ has at least one tie between the positive actual state group and the negative actual state group. Statistics may be biased. ^a^Group = control; ^b^under the nonparametric assumption; ^c^null hypothesis: true area = 0.5. VBQ threshold: 2.85; sensitivity: 91.7%; specificity: 23.5%. VBQ: vertebral bone quality; ROC: receiver operating curve.

Area under the curve^a^				
Test result variable(s)			Confidence level	
Area	Std error^b^	Asymptotic sig^c^	Upper bound	Lower bound
0.446	0.076	0.487	0.298	0.594

Based on the T-score, there was no significant difference in the prevalence of osteoporosis between the degenerative and control groups (18.5% vs. 24.1%, P = 0.46). Based on the VBQ threshold score of 3.25, the prevalence of osteoporosis in the degenerative group was significantly higher (55.5% vs. 40.9%, P = 0.025).

## Discussion

Osteoporosis is becoming a growing public health concern due to the aging population [1]. Since many people do not experience noticeable symptoms before a fracture, the condition often goes undiagnosed until it is too late. According to an International Osteoporosis Foundation survey, one of the main reasons for this is the limited opportunity for diagnosis before a fracture occurs, leading to underdiagnosis and under-treatment. In a study by Lewiecki et al., it was found that only 10%-53% of BMD measured in postmenopausal women over 65 years of age after a fragility fracture met the diagnostic criteria for osteoporosis [7]. Early detection and proactive screening could help reduce fractures by allowing for timely intervention and preventive measures [8].

The most widely utilized tool for the diagnosis of osteoporosis is DEXA. It provides a two-dimensional measurement of bone density per area (in grams per square centimeter), which can be influenced by bone size, potentially leading to an overestimation of fracture risk in shorter individuals. Additionally, DEXA is sensitive to degenerative changes, and people with significant degeneration may show higher bone density, which could underestimate their actual fracture risk. Other factors, like aortic calcification, spinal abnormalities, or changes after spinal surgeries, can also affect bone density readings [9].

In osteoporotic bones, there is increased adiposity, which results in increased signal on T1, and this raises the possibility of utilizing MRI as a tool for predicting bone quality [10]. Ehresman et al. [11] formulated the VBQ score, which is normalized to each patient using cerebrospinal fluid (CSF). It has a consistent composition across individuals, making it suitable for use across various MR scanners.

In our study, we found a statistically significant negative correlation between VBQ score and BMI in both the degenerative and control groups. These findings are consistent with those of Zhao et al. [12]. BMI has a complex and paradoxical relationship with the VBQ score. An increase in body mass index (BMI) increases static mechanical compliance, which leads to higher axial mechanical pressure and changes in bone structure, making bones stronger with a lower VBQ score. However, obesity can stimulate mesenchymal stem cells to differentiate into adipocytes (fat cells). Excessive accumulation of bone marrow adipocytes disrupts osteocyte activity and reduces bone turnover. Additionally, the proliferation of adipocytes in bone marrow promotes the release of pro-inflammatory substances, which increase osteoclast activity while inhibiting osteoblast differentiation leading to higher VBQ scores.

In our study, the patients were dichotomized into degenerative and control groups and the difference in correlation between DEXA measures and VBQ scores in the two study groups was analyzed. With increasing age, the prevalence of both osteoporosis and degenerative changes increases [13]. The combination of these has counter-active effects on DEXA. In our study, there was a positive correlation between age and VBQ score in both study groups (P < 0.01). Though the mean age of the degenerative group was higher than the control group, DEXA measures were found to be higher in the degenerative group. The weaker negative correlation between the VBQ score and DEXA measures in the degenerative group is likely due to the overestimation of bone density by DEXA. Conversely, the stronger negative correlation in the control group may indicate that the VBQ score more accurately reflects bone quality [14]. 

Based on our study findings, a VBQ score between 3 and 3.55 can be used as a cut-off for osteoporosis with reasonable sensitivity and specificity ranging between 85.7-71.4% and 47.7-81.8%, respectively. In a meta-analysis conducted by Chen et al. on eight worldwide studies including 999 patients, the VBQ score threshold ranged between 2.39 and 3.08 [5]. The difference in threshold between studies could be due to several factors, such as race, age, sex of the study population, physiological factors affecting bone composition, such as hyperlipidemia, which in turn affects VBQ score, scanner type used, etc.

Most of the MRI lumbar spine scans done in our routine practice are for lumbar disc disease and pre-operative assessment. Bone quality has great prognostic implications on the success of surgery [14]. Therefore, it is essential to accurately diagnose osteoporosis preoperatively and take preventative measures, such as utilizing bone cement screws, during surgery to help reduce the likelihood of mechanical complications postoperatively [5]. Extrapolating our study results to patients undergoing pre-surgical MR imaging for lumbar spondylosis, we can assess the VBQ score in such patients and alert the referring doctor if the score is above three so that further assessment with DEXA may be needed.

To our knowledge, this is the first study done in the Indian population with a reasonable sample size exploring the value of VBQ score in predicting bone quality. This study has a few limitations. It is a retrospective study done in a single center, including only patients of Indian ethnicity. We have not included the presence of aortic calcification to qualify for categorization into the degenerative group; however, it may have spuriously caused increased DEXA measures. Most of the patients in our study are women, and hence, sex selection bias might have interfered with our results. We have not evaluated the correlation between the DEXA measures from other sites, such as the femoral neck and VBQ score. We have not considered the influence of the patient’s lipid profile on the VBQ score.

## Conclusions

MRI-based VBQ score is a simple, easy-to-calculate tool that does not require exposure to ionizing radiation to analyze bone quality. VBQ scores potentially overcome the shortcomings of DEXA for diagnosing osteoporosis, especially in older patients with more degenerative changes, where setting DEXA may give spurious results. VBQ score may be used as a supplementary tool along with DEXA. However, MRI is an expensive investigation and its utility as a diagnostic tool for osteoporosis in the absence of other indications such as degenerative disc disease may not be justified. Further studies with larger sample sizes are required to formulate a VBQ score threshold cut-off value for osteoporosis diagnosis.
